# Conventional chemotherapy for acute myeloid leukemia: a Brazilian experience

**DOI:** 10.1590/S1516-31802000000600005

**Published:** 2000-11-01

**Authors:** Kátia Borgia Barbosa Pagnano, Fabiola Traina, Tatiana Takahashi, Gislaine Borba Oliveira, Marta Soares Rossini, Irene Lorand-Metze, Afonso Celso Vigorito, Eliana Cristina Martins Miranda, Cármino Antonio De Souza

**Keywords:** Acute myeloid leukemia, Chemotherapy, Bone marrow transplantation, Survival, Leucemia mielóide aguda, Quimioterapia, Transplante de medula óssea, Sobrevida

## Abstract

**CONTEXT:**

Young patients affected by acute myeloid leukemia (AML) achieve complete remission (CR) using conventional chemotherapy in about 55-85%. However, 30% of patients fail to achieve CR and the remission duration is often only about 12 months. More intensive treatment after CR seems to be necessary in order to maintain CR and obtain a definitive cure. In Brazil, few reports have been published on this important subject.

**OBJECTIVE:**

The aim of this study was to describe a Brazilian experience in the treatment of "*de novo*" acute myeloid leukemia (AML) in younger adult patients (age < 60 years).

**DESIGN:**

Retrospective analysis.

**SETTING:**

University Hospital, Hematology and Hemotherapy Center, State University of Campinas, Brazil.

**PARTICIPANTS:**

Newly diagnosed cases of *"de novo*" AML in the period from January 1994 to December 1998 were evaluated retrospectively, in relation to response to treatment, overall survival (OS) and disease free survival (DFS). Cases with acute promyelocytic leukemia (APL) were also included in this analysis.

**RESULTS:**

On the basis of an intention to treat, 78 cases of AML, including 17 cases of APL, were evaluated. The overall median followup was 272 days. The complete remission (CR) rate was 63.6% in the AML group (excluding APL) and 78% in the APL group. The 5- year estimated disease-free survival (DFS) was 80% for the APL group and 34% for the AML group (*P* = 0.02). The 5-year estimated overall survival (OS) was 52% for the APL group and 20.5% for the AML group, respectively (*P* = NS). Relapse was observed in 12/39 (30.7%) patients with AML and 1/11 (9%) with APL.

**CONCLUSIONS:**

These results are similar to those reported in the literature. However, relapse and mortality rates remain high, and a search for more aggressive strategies in order to prevent relapse is recommended.

## RESUMO

**CONTEXTO:**

Pacientes adultos com diagnóstico de leucemia mielóide aguda (LMA) obtém remissão completa (RC) com quimioterapia convencional em cerca de 55-85% dos casos, e a sua duração é de aproximadamente 12 meses. Cerca de 30% dos pacientes não atingem a RC. Para manter a RC e obter cura definitiva parece ser necessário intensificar o tratamento após a RC. No Brasil, há um pequeno número de artigos publicados a esse respeito.

**OBJETIVO:**

Descrever a experiência brasileira no tratamento de leucemia mielóide aguda "de novo" em jovens e adultos com idade inferior a 60 anos.

**TIPO DE ESTUDO:**

Análise retrospectiva.

**LOCAL:**

Hospital das Clínicas e Hemocentro da Universidade Estadual de Campinas.

**PARTICIPANTES:**

Foram estudados, retrospectivamente, casos novos de LMA entre 1994 e 1998, com relação à resposta ao tratamento de quimioterapia, sobrevida global e sobrevivência livre de doença.

**RESULTADOS:**

Entraram no estudo 78 pacientes de LMA, incluindo 17 casos de leucemia promielocítica (LPM). A média de seguimento foi de 272 dias. A taxa de RC foi de 63,6% nos pacientes com LMA excluindo-se os pacientes com LPM, e 78% nos pacientes com LPM. A sobrevida livre de doença em 5 anos foi de 80% nos pacientes com LPM e 34% para os pacientes com LMA (*P*=0,01). A sobrevida global foi de 52% e 20,5% para os pacientes com LPM e LMA respectivamente (*P*=NS). Recidiva foi observada em 12/39 (30,7%) dos pacientes com LMA e 1/11 (9%) na LPM.

**CONCLUSÕES:**

Esses resultados são semelhantes aos encontrados na literatura, entretanto, o número de recidivas e a mortalidade mantém-se alta, recomendando-se pesquisa de estratégias mais agressivas para prevenir recidivas.

## INTRODUCTION

During the 1980's and 1990's an increasing majority of patients with AML achieved CR and the proportion of patients remaining in permanent remission also improved.^1,2^ Intensified induction therapy may affect the long term survival without an apparent effect on the initial response rate.^3^ CR rates in previously untreated patients with *de novo* AML using High Dose Ara-C (HDAC) have been reported to be 50-80%.^4,5^

The general trend in the therapeutic strategy has invariably been aimed towards more aggressive treatment administered as soon as CR is achieved. The reason for this has been to provide a maximum of antitumor effect, a so-called consolidation or intensification treatment.^6^

Three intensive consolidation strategies are currently proposed for younger adults with AML in first CR: allogeneic or autologous bone marrow transplantation (BMT) or intensive consolidation chemotherapy.^7,8^ For patients who are younger than 45, myeloablative treatment with allogeneic BMT, from an identical HLA sibling donor, has become an accepted treatment, when a donor is available. However, allogeneic BMT carries substantial procedural risk related to mortality and morbidity, resulting in a 5-year overall survival of around 50%.^8^ Autologous stem cell transplantation offers the possibility of performing the same myeloablative regimen in patients without a compatible donor and without the risks associated with graft-versus-host-disease (GVHD).^7^

Results from randomized studies comparing chemotherapy alone to allogeneic and autologous bone marrow transplantation have become available.^7-11^ Better results are seen in APL, a distinct subtype of AML, in which a balanced reciprocal translocation between chromosomes 15 and 17 results in the union of portions of the promyelocytic leukemia gene with the gene for retinoic acid receptor alpha. The disease typically presents with a life-threatening hemorrhagic diathesis, which worsens by cytotoxic chemotherapy, with early hemorrhagic deaths of about 10-20%. The use of all-trans-retinoic acid (ATRA) followed by anthracycline in the induction therapy of APL has improved the outcome, with the CR rate reaching more than 90% and reducing the incidence of relapse.^12,13,14^

The aim of this study was to describe a Brazilian experience in the treatment of adult patients affected by AML, including APL, providing a basis for more intensive approaches in the future.

## METHODS

### Patients.

Patients under 60 years of age, with previously untreated "*de novo*" acute myeloid leukemia (AML) diagnosed and treated between 1994 and 1998 at the State University of Campinas were enrolled in this study. The diagnosis was assessed by bone marrow (BM) aspiration showing at least 30% of blasts, or by a bone marrow biopsy, in the case of associated myelofibrosis. Each case was classified according to the French-American-British (FAB) System.^15,16^ Patients with a previous history of myelodysplasia, myeloproliferative disease and previous chemotherapy or radiotherapy treatment were not included in this study.

### Leukemia diagnosis.

For the classification we used the procedures recommended by Scott, et al.^23^ We used conventional cytochemistry techniques for the classification of AML: Sudan Black, PAS and unspecific esterase, with and without NaF inhibition. Cases considered to be Sudan Black negative were studied by flow cytometry with the following antibodies: CD45, CD10/CD19, CD3/DR, CD7/CD33, CD14/CD34, CD13 and myeloperoxidase (MPO). Immunocytochemistry in slides was performed with antibodies to glycophorin, CD41 and CD62 when necessary. APL was classified by morphology. Cytogenetic studies were available for only two APL patients: one presented t (15.17) and PCR positive for the translocation PML/RARα. The other one had variant microgranular morphology and had a normal karyotype.

### Treatment.

The induction treatment consisted of 1 or 2 cycles of TAD-9, as described elsewhere.^1^ Bone marrow aspiration was performed between the 14^th^ and 21^st^ day after the last day of chemotherapy. Patients who did not achieve complete remission (CR) after two cycles of TAD-9 were treated with a salvage regimen, named MEC.^17^ Patients who achieved CR were submitted to two consecutive consolidation cycles, one with HDAC plus daunorubicin (ARAC 2g/m^2^ IV twice a day on days 1-4 and daunorubicin 45 mg/m^2^ on days 5-7), followed by one cycle of TAD-7, as described elsewhere.^1^ After these 2 cycles, patients were submitted to maintenance treatment for 24 months, with monthly cycles of ARAC 100 mg/m^2^ SC twice a day on days 1-5, alternating monthly cyclophosphamide, daunorubicin and thioguanine.^2^

A search for an HLA identical donor was performed for all patients under 50 years of age, and allogeneic BMT was proposed to patients with an HLA-identical sibling donor available (except to APL patients).

Patients affected by APL received an ATRA-containing regimen as induction treatment, according to initial leukometry. Patients with WBC = 5×10^9^/L were treated with ATRA. Patients with WBC between 6×10^9^/L and 10×10^9^/L were treated with ATRA and TAD-9 and patients with WBC >10×10^9^/L at diagnosis received only conventional chemotherapy as induction treatment (TAD-9). One patient was treated with ATRA and daunorubicin as induction. ATRA was interrupted and chemotherapy added if patients presented ATRA syndrome and/or leukocytosis. The post remission therapy for APL patients was the same used for the other AML subtypes. No allogeneic BMT was performed in APL patients during the first CR.

### Response criteria.

CR was defined as more than 1.0×10^9^/L granulocytes and more than 100×10^9^/L platelets in peripheral blood, and normocellular BM containing less than 5% of blasts cells. Patients who did not fulfill the above criteria were considered nonresponders.

### Statistical methods.

Analysis was based on status of the patients on April 20^th^ 1999, based on the last follow-up. Overall survival (OS) was defined from the date of diagnosis and beginning of chemotherapy until death or last follow-up. Disease free survival (DFS) was calculated from the date of first complete remission until the date of death, first relapse or last follow-up in continuous CR. Survival curves were calculated by the Kaplan-Meier method and differences between the curves were analyzed with the log-rank test.^18^ The patients were divided into the AML group (n=61) and APL group (n=17). The comparison between WBC number on diagnosis was based on descriptive analysis using the Mann-Whitney test.

## RESULTS

Between January 1994 and December 1998, 87 patients with age below 60 years old were diagnosed as AML in our hospital. However, nine patients were not evaluated because of early death before starting chemotherapy. Among the 78 evaluated patients, 61 (78.2%) patients were classified as AML group (nonAPL) and 17 (21.8%) were classified as APL. In the AML group, 31 patients were males and 30 females and the median age was 32 years (14-59). In the APL group, 7 patients were males and 10 females, with a median age of 33 years (16-54). The median number of white blood cells (WBC) at diagnosis was 20 × 10^9^/L (0.4214) for the AML group and 3.3 × 10^9^/L (0.7-49) for the APL group (*P* = 0.004) (Table 1). The clinical and laboratory characteristics are shown in Table 1. The most common FAB subtypes were M4, M2 and M3 (32%, 21.8% and 21.8%, respectively) (Table 2). During the analysis period, no cases of M7 subtypes were diagnosed. The overall median follow-up was 254 days (1-1842) and 540 days (4-1580) for the AML and APL groups, respectively.

**Table 1:** French-American-British classification of 78 patients affected by acute myeloid leukemia

	N	%
M0	2	2.5
M1	6	7.8
M2	17	21.8
M3	17	21.8
M4	25	32
M5	6	7.8
M6	1	1.3
M7	0	0
not classified*	4	5
Total	78	

* 3 biphenotypic AML, one case of granulocytic sarcoma.

**Table 2:** Clinical and laboratory characteristics of patients with AML

	AML group (n=61)	APL group(n=17)
median age	32 (14 to 59)	33 (16 to 54)S e x
(M/F)	31/30	7/10
Hb (g/dl)	8.3 (4.5 to 15)	8.0 (5 to 12)
WBC x10^9^/L	20 (0.4 to 214)	3.3 (0.7 to 49)
% blasts peripheral blood	60.5 (0 to 98)	15 (3 to 91) %
blasts bone marrow	76 (30 to 99)	75.5 (38 to 99)
Platelets x10^9^ /L	42 (2 to 247)	18 (2 to 146)
median follow-up (days)	261.5 (1 to 1842)	419 (4 to 1580)

AML - acute myeloid leukemia; APL - acute promyelocytic leukemia; M - male; F - female.

### Response.

At the end of our observation, 33/78 patients (42.3%) were alive; 32 (41%) in CR and 1 (1.2%) with refractory disease after relapse; 45/78 (57.6%)

patients died. *AML group*: 30 out of 61 (49%) patients reached CR with one cycle of TAD-9. Thirteen out of 61 did not achieved CR using one cycle of TAD-9, and 5 of them (8.1%) achieved CR after the second cycle of TAD- 9. Three patients (4.9%) achieved CR after salvage chemotherapy with MEC (2 patients) or HDAC (1 patient). One patient (1.6%) received as an induction treatment low dose Ara-C due to poor performance status at diagnosis and achieved CR. A total of 39 patients achieved CR (63.6%). Two patients were submitted to allogeneic bone marrow transplantation, while in first CR and remained in CR. Four patients died during consolidation therapy while in CR. Twelve out of 35 (34.2%) patients relapsed, after a median time of 287.5 days (50-1265) from first CR (AML group). *APL patients*: Seven patients with WBC ≤ 5×10^9^/L were treated initially with ATRA. TAD-9 was associated in 7 cases with a rise in WBC and/or ATRA syndrome. Eleven out of 14 (78%) patients who used an ATRA-containing regimen achieved CR. Three patients were treated with conventional chemotherapy alone and died during induction. One out of 11 (9%) relapsed, after 22 months in CR, during maintenance treatment.

### Survival.

**Figure 1:**
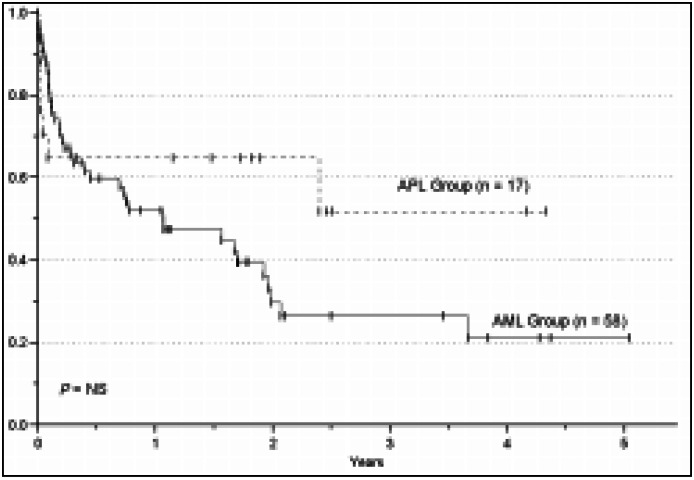
Overall survival in patients affected by AML.

**Figure 2:**
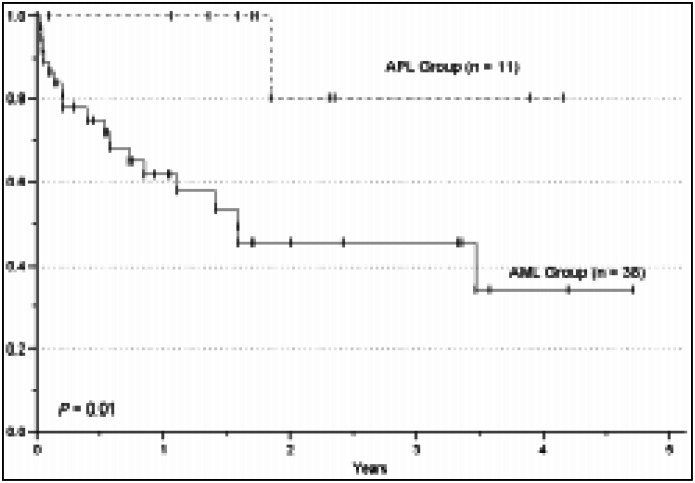
Disease free survival in patients affected by AML.

The median survival for alive patients was 409 days (67-1842) for the AML (non-APL) group (n=22) and 677 days (118-1580) for the APL group (n=10). The estimated OS for all patients (AML and APL groups) was 27% over 5 years. The 5-year estimated OS was 20.5% and 52% for AML and APL groups, respectively (*P* = NS). However, the 5-year estimated DFS was significantly higher in the APL (80%) compared to the AML group (34%) (*P* = 0.01). The overall survival and disease free survival curves for the two groups of patients are shown in Figures 1 and 2, respectively.

### Causes of death.

AML group: 21 out of 61 (34.5%) patients died during induction treatment. 13 out of 21 (61.9%) died due to infection, 2 out of 21 (9.5%) due to CNS bleeding, 1 out of 21 (4.8%) due to leukostasis, 1 out of 21 due to acute GVHD, and 4 out of 21 (19%) due to unknown causes. After consolidation therapy, 4 out of 39 patients (10.2%) died during the aplastic phase, due to infection; 3 of them after the first cycle of consolidation (HDAC) and one after the second cycle of consolidation (TAD-7). Eleven out of 35 patients (31.4%) died after relapse. One out of 11 (9%) patients died of bleeding before receiving reinduction treatment. Nine out of 11 (82%) died due to infection, after reinduction chemotherapy. One out of 11 (9%) was submitted to allogeneic BMT and died due to progressive disease and central nervous system bleeding. None of the relapsed patients achieved CR using salvage therapy. No patients died during maintenance treatment, while in CR. *APL group:* There were 7 deaths in this group. Three out of 7 (44%) patients died during TAD-9 induction, two due to disseminated intravascular coagulation (DIC) and one due to infection. Among the patients treated with an ATRA-containing regimen, 2 out of 7 (28%) patients died due to ATRA syndrome and one out of 7 (14%) due to infection. One of the 7 (14%) patients had a late relapse, 22 months after starting CR and died during the aplastic phase post-reinduction therapy, due to infection.

## DISCUSSION

In this retrospective study, we analyzed the results of conventional treatment for patients with AML, age < 60 years, treated at the University of Campinas. There are few Brazilian studies published concerning results of treatment in AML.^19^ For this age group, there are many reports referring to CR rates between 55% to 85%, with prolonged DFS, and cures in about 25%-30%.^4^

We used TAD-9 as induction therapy. The CR rate after the 1^st^ cycle was 49%. The second cycle of TAD-9 brought little improvement to the overall CR (8.1%). In addition, 4.9% of patients achieved CR with salvage regimens. The overall CR rate was 63.6%, similar to those reported in the literature.^2,4,5^ With standard induction regimens (3 days of anthracycline and 7 days of cytarabine), approximately 30% of patients still fail to achieve CR and the remission duration is often around 12 months.

HDAC has been successfully used in a number of combinations in relapsed patients and as post-remission or consolidation therapy.^9,10,11^ We used one cycle of HDAC for consolidation and did not observe high toxicity and mortality. Thus, this regimen was demonstrated to be feasible in our patients. Although many different schedules have been used, including consolidation with or without prolonged maintenance, about 25-30% of patients will remain in CR for 5 years. However, the maintenance of remission is still a challenge. We observed relapse in 34.7% of the AML group, half of them occurring during the first year post-remission. Our maintenance program was not able to sustain CR in a large number of patients. The 5-year estimated overall survival for the AML group was 20.5%, similar to that reported in the literature.^2,4,5^

Recently, new approaches have been proposed to intensify AML treatment, with the purpose of decreasing relapse incidence, leading to a better OS and DFS. The first large randomized study comparing allogeneic BMT, autologous BMT and intensive consolidation with HDARAC was published by the EORTC and GIMEMA groups.^20^ In this study, BMT resulted in a better DFS than conventional chemotherapy. In the GOELAM study, the 3 groups presented similar results. However, this group used higher doses of ARAC and idarubicin instead of daunorubicin. In this study only 32% of patients in CR could be randomized between autologous BMT and consolidation with chemotherapy. Moreover, allogeneic BMT was performed without previous intensive consolidation, resulting in a large number of relapses after bone marrow transplantation. The MRC AML 10 trial study showed a better OS and fewer relapses after 4 cycles of chemotherapy followed by autologous BMT. However, in this trial HDARAC was not used. Based on this study, 3 prognostic groups were described according to cytogenetics, FAB classification and response to the first cycle of induction.^21^ The group with favorable prognosis consisted of patients with APL or favorable karyotype (inv 16, t(15-17), t(8-21); the standard risk group (neither good nor poor) and the poor risk group were those with adverse karyotype or > 15% of blasts after the first course of induction. However, except for APL, there is still no consensus in the literature for differentiating AML treatment according to cytogenetic characteristics. In our study, it was not possible to evaluate cytogenetics in most of the cases, so we could not stratify the patients into risk categories according to this criterion.

The treatment of the APL group using chemotherapy alone was shown to be inadequate, and high rates of mortality due to DIC were observed. The use of all-trans-retinoic acid (ATRA) followed by anthracycline, in the induction therapy of APL, has improved the CR rate to greater than 90% and reduced the incidence of relapse.^12,13,14^ The addition of ATRA to chemotherapy during induction of APL has brought a great improvement in terms of CR and DFS, also in our patients. DFS was longer in APL (80%) than in the AML (non-APL) group (34%) (*P* = 0.02). The difference in terms of OS between the 2 groups was not significant, probably due to the small number of APL patients enrolled. Once CR was achieved, relapse was a rare event in this group. We did not observe the high induction mortality rates described by Pulcheri, et al.^19^

Except for APL, allogeneic bone marrow transplantation from an HLA sibling donor is the treatment of choice, despite the transplant-related morbidity and mortality. In our study, patients transplanted in the first CR had better results than those with refractory disease. However, few patients have an available donor. More intensive chemotherapy followed by autologous BMT, or more intensive consolidation, should probably reduce the relapse risk and prolong overall survival.

We may therefore conclude that the overall results achieved at our Institution represent what is expected in the literature for conventional chemotherapy in AML and APL.

Based on this previous experience, we are introducing a new prospective approach to AML treatment: intensification of consolidation with a second HDAC, instead of TAD-7, and a randomized study comparing autologous bone marrow transplantation versus maintenance treatment, for young patients who do not have identical sibling HLA donors available. With this novel strategy, we expect to offer a better chance of cure for these patients.
